# Extent and Predictors of Delays in Diagnosis of Cervical Cancer in Addis Ababa, Ethiopia: A Population-Based Prospective Study

**DOI:** 10.1200/JGO.19.00242

**Published:** 2020-02-28

**Authors:** Nebiyu Dereje, Adamu Addissie, Alemayehu Worku, Mathewos Assefa, Aynalem Abraha, Wondemagegnehu Tigeneh, Eva Johanna Kantelhardt, Ahmedin Jemal

**Affiliations:** ^1^ School of Public Health, Wachemo University, Hosanna, Ethiopia; ^2^ Department of Preventive Medicine, School of Public Health, Addis Ababa University, Addis Ababa, Ethiopia; ^3^ Department of Oncology, School of Medicine, Addis Ababa University, Addis Ababa, Ethiopia; ^4^ Department of Gynaecology and Institute of Medical Epidemiology, Biostatistics and Informatics, Martin-Luther-University, Halle-Wittenberg, Germany; ^5^ Surveillance and Health Services Research, American Cancer Society, Atlanta, GA

## Abstract

**PURPOSE:**

A substantial proportion of cervical cancers are diagnosed at advanced stage in Ethiopia. Therefore, the aim of this study was to determine the extent and predictors of delays in cervical cancer diagnosis in Addis Ababa.

**PATIENTS AND METHODS:**

We prospectively recruited 231 patients with cervical cancer diagnosed from January 1, 2017, to June 30, 2018, in 7 health facilities in Addis Ababa, representing 99% of all cervical cancers recorded in the Addis Ababa population-based cancer registry. A structured questionnaire on patients’ experience was administered face to face by trained interviewers. Health-seeking intervals > 90 days (date from recognition of symptoms to medical consultation) and diagnostic intervals > 30 days (dates from medical consultation to diagnostic confirmation) were categorized as delayed. Factors associated with these delays were assessed using multivariable binary logistic regression models.

**RESULTS:**

The median health-seeking and diagnostic intervals for patients with cervical cancer in Addis Ababa were 10 and 97 days, respectively. Approximately one quarter of the patients were delayed in seeking medical consultation, and three fourths of the patients had delayed diagnostic confirmation. Factors associated with health-seeking delays included poor cervical cancer awareness, practicing of religious rituals, and waiting for additional symptoms before visiting a health facility. Factors associated with diagnostic delays included first contact with primary health care units and visits to ≥ 4 different health facilities before diagnosis.

**CONCLUSION:**

A considerable proportion of patients with cervical cancer in Addis Ababa have delays in seeking medical care and diagnostic conformation. These findings reinforce the need for programs to enhance awareness about cervical cancer signs and symptoms and the importance of early diagnosis in the community and among health care providers.

## INTRODUCTION

Cervical cancer is one of the most preventable causes of death resulting from cancer.^1-3^ However, in Ethiopia, as in several parts of sub-Saharan Africa, it remains a leading cause of death resulting from cancer in women,^4-6^ largely because of a lack of screening, inadequate therapy,^6-8^ and poor knowledge about the disease.^9,10^ Screening for cervical cancer not only detects cancer at earlier stages but also allows detection and removal of precancerous lesions.^7^ According to previous studies, however, only 0.6% of women in Ethiopia have ever had cervical cancer screening.^6^ As a result, a substantial proportion of patients in Addis Ababa, the capital city of Ethiopia, and other parts of the country are diagnosed at later stages of the disease, when the choice of treatment is limited and the chance of survival is poor.^11^ In addition to a lack of screening, poor awareness of the disease in the community and a less developed health system contribute to late presentation of cervical cancer in Addis Ababa and other parts of Ethiopia.^9,10^

**CONTEXT**

**Key Objective**
Early detection is important for the prevention and control of cervical cancer. However, the extent of delay in receipt of diagnostic confirmation among patients diagnosed with cervical cancer in Addis Ababa is unknown. Also, unknown are patient and provider factors contributing to this delay.
**Knowledge Generated**
Delays in receipt of diagnostic confirmation for cervical cancer was substantial in Addis Ababa. This was strongly associated with poor knowledge of cervical cancer among patients, use of traditional medicine, primary health care unit as the point of entry to the healthcare system, and visits to more than four different health facilities before diagnostic confirmation.
**Relevance**
The findings of this study underscore the need for programs to focus on enhancing awareness of cervical cancer among the general population and health care providers and to decentralize cervical cancer diagnostic services to the primary health care level in Addis Ababa, Ethiopia.

Only two studies have examined delays in disease presentation among patients with cervical cancer in Ethiopia. These studies both found that delays were substantial and were associated with a lack of disease awareness and other sociodemographic factors.^12,13^ Both studies, however, were based solely on patients seen at a single health facility (Tikur Abessa Specialized Hospital [TASH]) and cannot be generalizable to the rest of Addis Ababa or Ethiopia. Several studies in other developing countries have also estimated the magnitude and predictors of delays in cervical cancer presentation, with more than half of patients in Morocco,^14-16^ Kenya,^17-20^ and Nepal^21,22^ have been found to experience patient-related or diagnostic delays.

In this study, we examined the extent of health-seeking (help-seeking) and diagnostic intervals among all patients with cervical cancer diagnosed from January 1, 2017, to June 30, 2018, in Addis Ababa, with health-seeking and diagnostic intervals defined according to the Aarhus model.^14,21,23^ We also examined patient- and provider-related factors associated with longer health-seeking and diagnostic intervals (delays).

## PATIENTS AND METHODS

### Study Setting and Design

Ethiopia has a 3-tier health care delivery system: primary-level health care delivered at health centers, secondary-level health care delivered at general hospitals, and tertiary-level health care delivered at specialized hospitals.^24^ There were 652 primary- and 33 secondary- or tertiary-level health care facilities in Addis Ababa in 2018.^25^ For our study, we recruited 234 women diagnosed with invasive primary cervical cancer from January 1, 2017, to June 30, 2018, in Addis Ababa attending 1 of 7 specialized health care facilities (TASH, St Paul, United Vision, Hallelujah, Leghar, Myungsung Christian Medical Center, and Bethezata) for their clinical care. According to data from the Addis Ababa population-based cancer registry,^26^ these patients represented all newly diagnosed cervical cancer cases in the city during the corresponding period, except for 3 patients who sought their clinical care abroad.

### Data Collection Tools and Procedures

A structured questionnaire was adapted from related studies to measure the experience (pathways) of patients with cancer from symptom recognition to diagnosis and treatment.^23,27,28^ The tool was developed in English and later translated into Amharic, the national language. The consistency of the translated questionnaire was checked by translation of the questionnaire back into English by an independent translator. Before administering the questionnaire, its contents were reviewed by experts (oncologists and gynecologists) to ensure validity. A face-to-face interview was conducted with each patient at recruitment or shortly thereafter by a trained nurse interviewer. Date of diagnosis confirmation, however, was based on information recorded in the medical record. The questionnaire was pretested for cultural appropriateness and clarity. To minimize recall bias, only newly diagnosed patients were included in the study. To aid collection of data on patients’ symptom recognition, the following three options (abnormal vaginal bleeding, abnormal vaginal discharge, and pain during sexual intercourse) were read to patients during the interviews. The study was approved by the institutional review board of Addis Ababa University, College of Health Sciences.

### Data Management and Analysis

Date of first symptom was defined in this study as the date when the patient noticed or recognized the primary symptom related to cervical cancer for the first time. Date of first consultation with a health care provider was defined as the first date at which a patient visited/consulted a health care provider after recognition of the first cervical cancer symptom by the patient. To facilitate the recall of the dates, different calendars and events (eg, holidays) were used during interviews. For patients who could not recall the exact dates (n = 6; 2.6%), an option was given to recall months and weeks, and then the middate of the week was used. Date of diagnostic confirmation, however, was obtained from the patient’s medical record.^14,23^

The Aarhus model^23^ was used to define the health-seeking and diagnostic intervals. Accordingly, the health-seeking interval was defined as the interval between the date of first symptom recognition by the patient and first date a patient visited/consulted a health care provider for the cervical cancer symptom. The health-seeking interval was considered delayed if it was > 90 days. The diagnostic interval was defined as the interval from the first date a patient visited/consulted a health care provider to the date of histologically confirmed diagnosis. The diagnostic interval was considered delayed if it was > 30 days.^14,21,23^

The type of health facility a patient first visited was categorized as a primary health care facility or secondary/tertiary health care facility. Primary health care facilities included health centers and private clinics, whereas secondary/tertiary health care facilities included government or private hospitals.^29^ Primary health care facilities are the entry point for seeking care in the Ethiopian health system; they provide basic preventive and curative services and are largely staffed by nurses, health officers, and general practitioners. In contrast, secondary/tertiary health care facilities are private or government hospitals, and they provide inpatient and ambulatory services.

Descriptive analyses were used to calculate summary statistics, and bivariable and multivariable binary logistic regression models were used to identify factors associated with patient (health-seeking) or diagnostic delays. Level of significance was set at *P* < .05 at a 95% CI, and odds ratio (OR) was used to quantify the strength of association for each of the variables.

## RESULTS

### Sociodemographic Characteristics of Participants

The median time interval from date of diagnostic confirmation to date of administration of first study interview for women with cervical cancer in our study was 33 days (interquartile range, 13-68 days). Age of the patients ranged from 23 to 86 years, with a mean of 52.6 years (standard deviation [SD], ± 13.17; 95% CI, 50.86 to 54.35 years). More than one third of the women were unable to read or write, and approximately two thirds of the women were housewives. Approximately two thirds of the women reported a family monthly income of < 3,201 Ethiopian birr (approximately US$100). Approximately 70% of patients paid their full medical bills out of pocket, and only 30% of patients were entitled to free full medical services for their cancer diagnosis (Table 1).

**TABLE 1:**
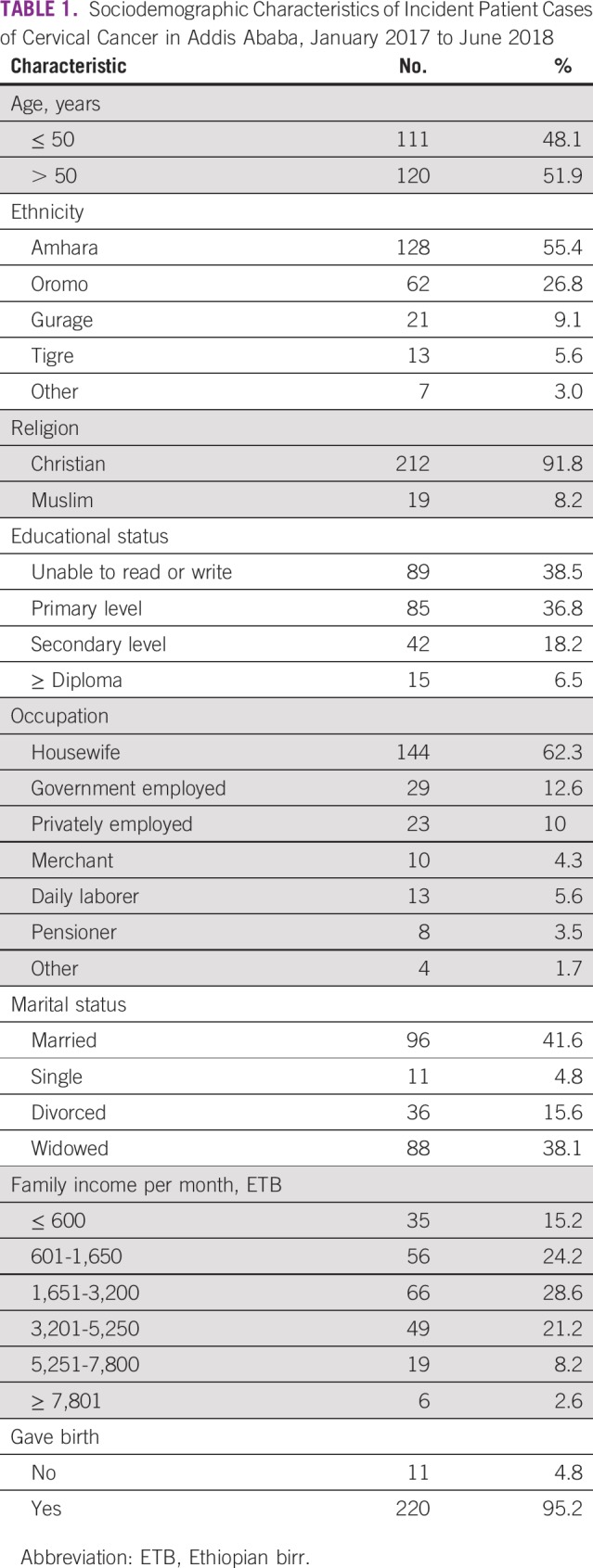
Sociodemographic Characteristics of Incident Patient Cases of Cervical Cancer in Addis Ababa, January 2017 to June 2018

### Cervical Cancer Awareness

Nearly two thirds (63.2%) of the patients had not heard of cervical cancer before their diagnosis. Only one tenth (10.4%) of the patients reported they had ever been screened for cervical cancer before diagnosis. Among those screened, a majority (91.7%) received the Papanicolaou test, and the remaining (8.3%) underwent visual inspection of the cervix with acetic acid.

### Patient Symptom Experience and Presentation

The most common first (primary) symptom experienced by the patients with cervical cancer was abnormal vaginal bleeding (69.7%; Table 2). A majority (71.9%) of the patients went to health facilities immediately (within 3 days) after recognition of symptoms. Moreover, during the prediagnosis period, 58% performed a religious ritual such as tebel (bathing in or drinking spring water, considered as holy) or prayer as a solution for their disease.

**TABLE 2:**
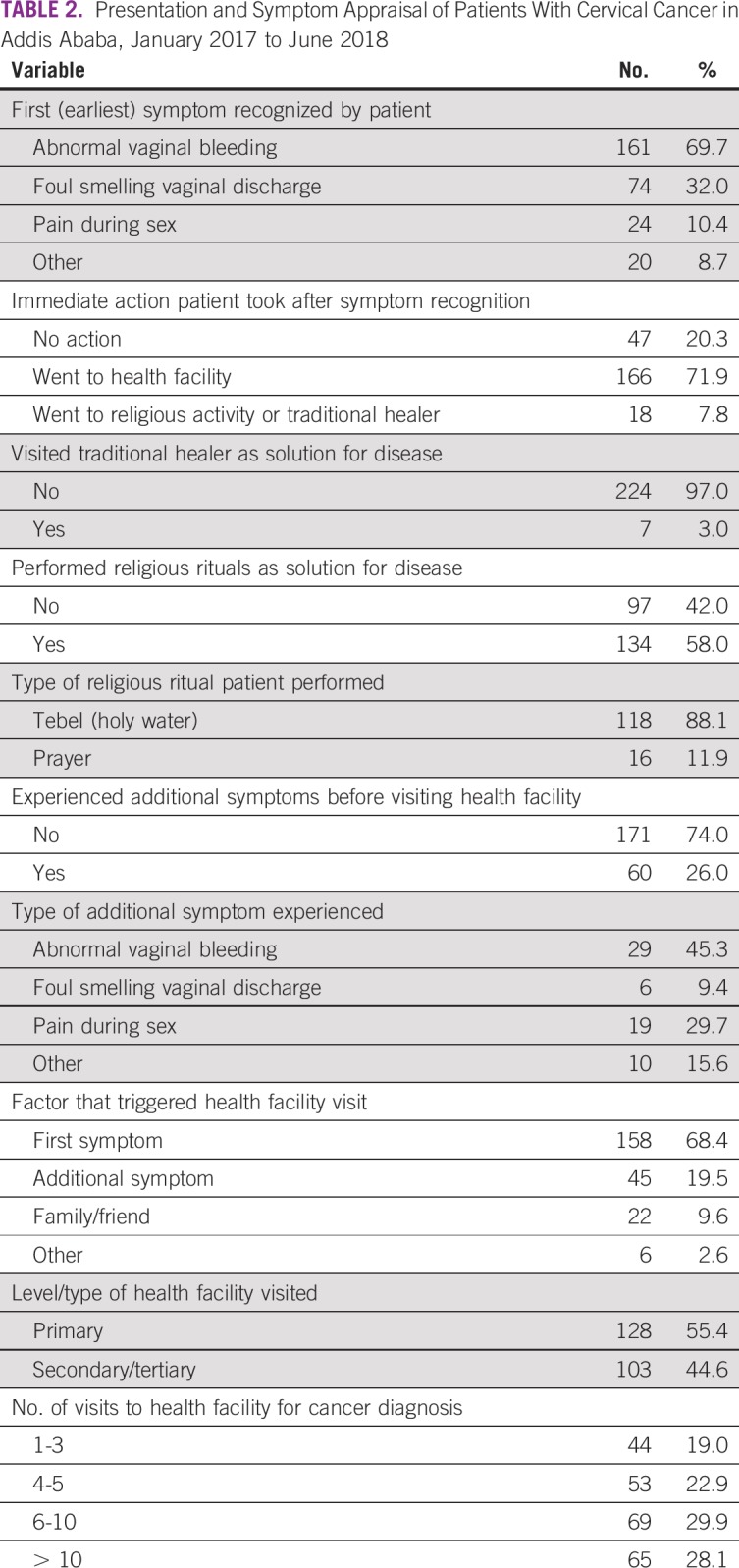
Presentation and Symptom Appraisal of Patients With Cervical Cancer in Addis Ababa, January 2017 to June 2018

Twenty-eight percent of the patients with cervical cancer experienced additional (second) symptoms, with abnormal vaginal bleeding as the most common second symptom, reported by 50% of these patients. Although a majority (68%) of patients were triggered by their first symptom to visit health care facilities, 22.5% of patients waited until they experienced additional symptoms.

With respect to the type of health facilities visited by patients and the frequency of such visits, more than half (55.4%) visited primary-level health care facilities. Patients visited on average 3 different health facilities (mean ± SD, 3.03 ± 1.34 days) before diagnostic confirmation, but 30% of the patients made a total of > 10 visits to health care facilities before diagnostic confirmation.

### Estimates of Health-Seeking Delays

The median health-seeking interval of the patients was 10 days (95% CI, 3 to 26 days). The health-seeking interval was > 90 days (delayed) for 23.4% (95% CI, 18.2% to 29.0%) of patients (Table 3). The two most common reasons given by patients for delays in seeking medical care were thinking that the symptom would go away by itself (38.9%) and not being bothered about the first symptom (29.6%).

**TABLE 3:**
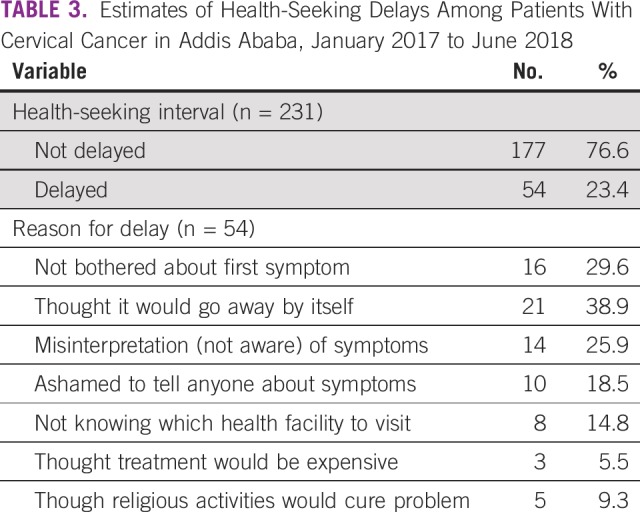
Estimates of Health-Seeking Delays Among Patients With Cervical Cancer in Addis Ababa, January 2017 to June 2018

### Factors Associated With Patient Health-Seeking Delays

In multivariable binary logistic regression analysis, patients’ health-seeking delays were significantly associated with a lack of cervical cancer awareness, practicing of a religious ritual as a solution for the disease, and waiting until experiencing additional symptoms (Table 4). The odds of delay in health seeking was 2.3 times higher in patients who had never heard of cervical cancer before diagnosis (adjusted OR [AOR], 2.3; 95% CI, 1.11 to 4.70) and in those who waited until they saw additional symptoms (AOR, 2.3; 95% CI, 1.096 to 4.90) compared with their counterparts. Moreover, the odds of health-seeking delays among patients who practiced a religious ritual as a solution for their cancer were 3 times higher than in those who did not (AOR, 3.3; 95% CI, 1.46 to 7.48).

**TABLE 4:**
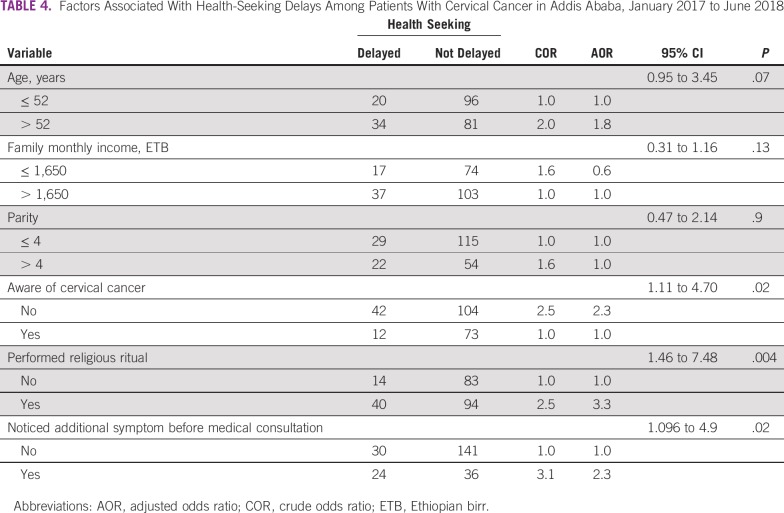
Factors Associated With Health-Seeking Delays Among Patients With Cervical Cancer in Addis Ababa, January 2017 to June 2018

### Estimates of Diagnostic Waiting Time (delays)

The median diagnostic interval for the patients was 97 days (95% CI, 81 to 123 days). More than three fourths (80.5%; 95% CI, 75.8% to 85.3%) of the patients with cervical cancer waited for > 30 days from their first health care provider consultation to histologically confirmed diagnosis of cervical cancer.

### Factors Associated With Diagnostic Delays in Cervical Cancer

In a multivariable binary logistic regression analysis, diagnostic delay was significantly associated with the level of first contacted health facility, number of different health facilities the patient visited for diagnosis, and total number of times a patient visited health facilities for diagnosis (Table 5). The odds of diagnostic delays among patients who contacted primary-level health facilities (health centers and private clinics) first were 2.6 times higher than among those who first contacted secondary- or tertiary-level health facilities (AOR, 2.6; 95% CI, 1.33 to 5.27). Likewise, the odds of diagnostic delays among patients who visited ≥ 4 different health facilities for their cancer diagnosis were approximately 3 times higher than among those who visited < 4 different health facilities (AOR, 2.7; 95% CI, 1.07 to 6.71). Similarly, the odds of diagnostic delays among patients who made > 5 visits to health facilities before receipt of histologic diagnostic confirmation were 2 times higher than among those patients who made < 5 visits (AOR, 2.2; 95% CI, 1.05 to 4.43).

**TABLE 5:**
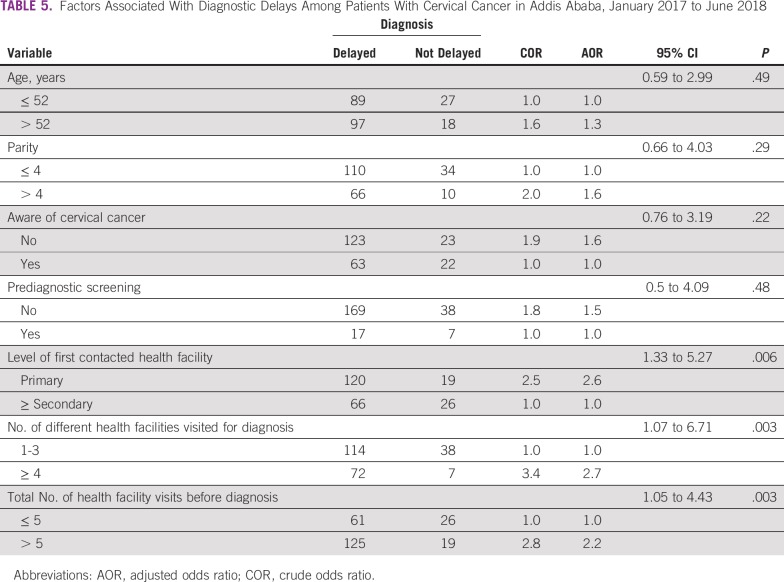
Factors Associated With Diagnostic Delays Among Patients With Cervical Cancer in Addis Ababa, January 2017 to June 2018

## DISCUSSION

Our principal findings were that approximately one fourth of women with cervical cancer in Addis Ababa delayed seeking medical consultation, and three fourths of the patients had delayed histologic diagnostic confirmation. We also found that health-seeking delays were strongly associated with a lack of cervical cancer awareness, practicing a religious ritual as a solution for the disease, and waiting for additional symptoms before visiting a health facility. Likewise, diagnostic delays were strongly associated with initial contact to primary health care units and visits to > 4 different health facilities before diagnosis. Notably, unlike previous findings from Ethiopia,^30,31^ our findings were based on a prospective population-based study and are likely more valuable in guiding public health policies to reduce the high burden of cervical cancer in the city and possibly in the country.

Our finding of the proportion of patients who delayed seeking medical consultation in Addis Ababa is considerably lower than findings from other developing countries, namely Kenya, Morocco, and Nepal, where approximately 60% of patients with cervical cancer experienced delays in seeking health care after symptom recognition.^14,16,19,21^ However, our finding is higher than those from Denmark and United Kingdom, where < 20% of patients experience health-seeking delays.^32,33^ These variations might be attributed to differences in the urban-rural composition of the study participants, educational attainment, health literacy, beliefs regarding traditional healers/religious rituals, and study periods. The studies from Kenya and Morocco had both urban and rural study participants, whereas participants in our study were predominantly from urban areas, where health literacy about cervical cancer is likely to be higher compared with that in rural areas. The relatively low proportion of patients with health-seeking delays in Addis Ababa may reflect better geographic access to the health facilities in Addis Ababa and high perception of the severity of the first symptom among the patients.

Our finding of strong associations between health-seeking delays and low awareness of cervical cancer and between health-seeking delays and waiting for additional symptoms are consistent with findings from other African countries, including Kenya and Morocco.^16,18-20^

The median diagnostic interval in Addis Ababa is considerably longer than those found in other developing countries, such as in Nepal (9 days),^21^ but it is slightly shorter than that reported in Morocco (110 days).^14^

It is interesting to note that our estimate of median diagnostic waiting interval is much longer than median patients’ health-seeking interval (97 *v* 10 days). The longer diagnostic time interval may reflect poor cervical knowledge among health care providers for prompt referral of patients and less developed diagnostic infrastructure including lack of pathologists in the city for timely diagnosis of the disease.^30,34-37^ Of note, patients with cervical cancer on average visited 4 different health facilities before receipt of histologically confirmed diagnosis. Approximately 9 of 10 patients with cervical cancer who visited > 4 different health facilities had diagnostic delays. Furthermore, approximately 86.3% of patients with cervical cancer who first contacted a primary health care unit were delayed in seeking medical consultation. This is likely because health care providers working at the primary care level are more likely to be nurses or health officers rather than medical physicians and thus less likely to be knowledgeable about cervical cancer for prompt referral of patients with cervical cancer symptoms.^37^ In addition, patients who seek medical care at the primary health care level are more likely to be those with lower educational attainment and poor awareness about cervical cancer.^13^ There are fewer than a handful of diagnostic facilities and only 2 pathologists for every 1 million people in the country,^38^ compared with 57 pathologists per 1 million people in the United States.^39^ These findings underscore the need for programs to enhance cervical cancer knowledge among health care providers working in health centers.

Our findings on the considerable delays in health seeking and in diagnosis of cervical cancer in Addis Ababa have significant implications for the success of the ongoing national cervical cancer screening program in the country.^29^ The health-seeking and diagnostic delays found in Addis Ababa are likely to be substantially lower than those expected in other parts of the country, because health literacy and access to primary and specialized care are better in Addis Ababa than in other parts of the country.^24^

A notable strength of our study is the use of the Addis Ababa population-based cancer registry to trace and recruit all patients with cervical cancer diagnosed in the city during the study period for the generalizability of the findings. Furthermore, this study was designed according to the Aarhus statement for international comparison of findings.^23^ Limitations of our study, however, include recall and social desirability biases related to dates of events and use of nonconventional care. In addition, our findings of patient and diagnostic time intervals are likely to be underestimated, because the study did not include those patients who did not access the health care system.

In conclusion, health-seeking and diagnostic delays are common among patients with cervical cancer in Addis Ababa, with one fourth and three fourths of patients experiencing health-seeking and diagnostic delays, respectively. These findings underscore the need for programs to enhance awareness of cervical cancer in the community (eg, churches and mosques) and among health care providers in health centers. There is also a need to improve the diagnostic infrastructure through training of more pathologists and procurement of diagnostic equipment.
